# A prospective pilot evaluation of vaginal and urine self-sampling for the Roche cobas 4800 HPV test for cervical cancer screening

**DOI:** 10.1038/s41598-018-27390-5

**Published:** 2018-06-13

**Authors:** Sang-Hyun Hwang, Hye Young Shin, Dong Ock Lee, Na Young Sung, Bomyee Lee, Do-Hoon Lee, Jae Kwan Jun

**Affiliations:** 1 Department of Laboratory Medicine, University of Ulsan College of Medicine and Asan Medical Center, Seoul, 05505 Republic of Korea; 2 National Cancer Control Institute, National Cancer Center, Goyang, 10408 Republic of Korea; 3 College of Nursing, Korea University, Seoul, 02841 South Korea Republic of Korea; 4 Center for Uterine Cancer, National Cancer Center Hospital, National Cancer Center, Goyang, 10408 Republic of Korea; 5 Kyung Hee University School of Medicine, Seoul, 02447 Republic of Korea; 6 Department of Laboratory Medicine, Center for Diagnostic Oncology, National Cancer Center Hospital, National Cancer Center, Goyang, 10408 Republic of Korea

## Abstract

This pilot study sought to evaluate the feasibility of utilizing vaginal self-swabs and urine samples for HPV-based cervical cancer screening in 700 women who had undergone conventional Pap smear screening via the national cervical cancer program in Korea. The cobas 4800 HPV test was utilized to detect HPV in the self-samples. Pap smear results revealed three cases of atypical squamous cells of undetermined significance, 649 cases of negative for an intraepithelial lesion or malignancy, and 48 non-specific inflammatory findings. High-risk HPV was detected in 6.7% of urine samples and 9.6% of vaginal self-swab samples. The overall agreement for HPV 16/18 between urine and vaginal self-swab samples was 99.1% (95%CI 98.1% to 99.6%). Colposcopic biopsy revealed one cervical intraepithelial neoplasia (CIN) 3 lesion, 12 CIN1 lesions, and 23 normal or chronic cervicitis lesions. In conclusion, urine and vaginal self-swab sampling was feasible and deemed a potential alternative for HPV detection in women who hesitate to participate in cervical cancer screening programs. Meanwhile, due to overall lower rates of abnormal cytology and sexual risk behaviors in Korea, a larger sample size than expected is needed to assess the sensitivity of CIN2+ detection via self-samples.

## Introduction

Cervical cancer is the seventh most commonly diagnosed cancer and the ninth leading cause of cancer death among females in Korea, with more than 3,633 incident cases annually and more than 892 deaths^1,2^. The age-standardized incidence of cervical cancer in 2013 was estimated at 9.5 per 100,000 in South Korea^1^ To address these rates, the National Cancer Prevention and Screening Program of Korea began to offer cervical cancer screening via cytology (Papanicolaou test or liquid-based cytology) to women aged over 30 years old every 2 years since 1999, extending to those over 20 years of age in 2016. However, cervical cancer screening with cervical sampling is invasive, time consuming, and suffers from poor screening attendance. In Korea, the participation rate in the national cervical cancer screening program was only about 51.5% in 2014^3^, although cervical cancer incidence has been increasing in younger age groups^4^. This suggest a need for new strategies to facilitate participation in the cervical cancer screening program in Korea.

Human papilloma virus (HPV) testing can be incorporated into screening programs as a triage for atypical squamous cells of undetermined significance (ASCUS) cytology, as a means to test for both HPV and cervical cytology (e.g., “cotesting”), and as a primary screening option for HPV^5–8^. Recently, evidence suggests that screening based on a validated test for oncogenic HPV DNA following an appropriate protocol is more effective than cytology-based screening for the prevention of invasive cancers of the uterine cervix^9–11^. As such, in 2014, the FDA approved the use of the cobas HPV test for primary cervical cancer screening for women over the age of 25 years, without the need for a concomitant Pap test^7^.

Self-sampling has been found to show high concordance with physician-acquired samples for HPV detection and to be readily acceptable to women^12,13^. As a noninvasive, self-sampling method, urine-based HPV DNA testing has been suggested to be of use in women who do not attend cervical cancer screening clinics^14^. Although non-invasive HPV testing may improve poor participant rates for cervical cancer screening in Korea, especially among young women, prospective cervical cancer screening with self-sampling for HPV in Koreans has not yet been extensively studied.

A few studies have compared HPV tests based on real-time qualitative PCR (RQ-PCR) of urine samples and cervical samples^15–17^. Previously, we showed that using vaginal swabs could be an alternative to collecting cervical samples for RQ-PCR-based HPV DNA testing^12^. We also demonstrated that fully-automated RQ-PCR systems could reduce the variability in HPV detection with non-invasive urine samples^18^.

This pilot study was conducted to obtain basic data necessary for larger-scale study to implement self-sampling in the national cervical cancer screening program in Korea. We also aimed to compare self-sampling methods, urine and vaginal self-swab samples, for HPV detection using the Roche cobas 4800 HPV Test (Roche_HPV; Roche Molecular Diagnostics, Pleasanton, CA, USA).

## Results

At enrollment, 12 women had missing or invalid test results, leaving 700 women available for analysis. The median age of the participants was 52 years (range 21–71 years). Among the study participants, cytology revealed three cases of ASC-US, 649 cases of negative for an intraepithelial lesion or malignancy (NILM), and 48 non-specific inflammatory findings. The self-sample results according to cytology are listed in Table 1.

**Table 1:** Self-sampling results according to cervical cytology results.

		Cervical cytology			Colposcopic Biopsy
		Normal	ASCUS	Inflammation	
**Vaginal self-swab (n = 694)**					
hrHPV negative (n = 627)		585	1	41	- chronic cervicitis (n = 3) - CIN1 (n = 2)
hrHPV positive (n = 67)	16/18	11	1	2	- normal (n = 4) - chronic cervicitis (n = 2) - CIN1 (n = 3) - CIN3 (n = 1)
	Other hrHPV	48	1	4	- normal (n = 2) - chronic cervicitis (n = 12) - CIN1 (n = 7)
**Urine (n = 679)**					
hrHPV negative (n = 634)		590	1	43	- normal (n = 3) - chronic cervicitis (n = 5) - CIN1 (n = 2)
hrHPV positive (n = 45)	16/18	9	1	0	- normal (n = 3) - CIN1 (n = 3) - CIN3 (n = 1)
	Other hrHPV	32	1	2	- chronic cervicitis (n = 12) - CIN1 (n = 6)

High-risk HPV (hrHPV) was detected in 6.7% of the urine samples and in 9.6% of the vaginal self-swab samples (Table 1). Among the vaginal self-swab samples, 14 were positive for HPV 16/18; 10 of the urine samples were positive for HPV 16/18. CIN3 was detected in both vaginal self-swab and urine samples showing HPV 16/18 positivity. Vaginal self-swab samples comprised a larger number of positive results for other hrHPV (n = 53) than urine samples (n = 35).

The overall agreement between urine and vaginal self-swab results for any hrHPV was 94.9% (95% CI 93.0 to 96.4%), with a kappa of 0.66, and the overall agreement for HPV 16/18 was 99.1% (95% CI 98.1% to 99.6%), with a kappa of 0.75. A statistically significant difference in detection of hrHPV was observed between urine and vaginal self-swab samples (p < 0.001, Table 2). Of 26 urine_negative/vaginal_positive samples, most were of other hrHPV type (5 cases were HPV16/18), and were all classified as NILM based on cytology.

**Table 2:** Comparison of urine and vaginal self-samples tested with the Roche HPV DNA test.

	HPV result		Vaginal self-swab		Kappa value	95% CI	P-value
			HPV DNA+	HPV DNA−			
Urine	HPV16 and/or HPV18	HPV+	9	1	0.7456	0.54–0.95	0.2188
		HPV−	5	658			
	Other hrHPV	HPV+	28	7	0.6481	0.52–0.78	0.0125
		HPV−	21	617			
	Any hrHPV	HPV+	37	8	0.6585	0.55–0.77	0.0030
		HPV−	26	602			

Among women with NILM or non-specific inflammation cytology, 33 underwent colposcopy (Table 3). Colposcopic findings revealed CIN1 in 11, chronic cervicitis in 16, and normal findings in six women. Among the women with chronic cervicitis (n = 16), 12 were positive for other hrHPV upon testing of their urine and vaginal self-swab samples; the four remaining women all had HPV-negative urine samples, with two being negative and the other two having HPV 16/18 positive vaginal self-swab samples. Cases with CIN1 (n = 11) and normal colposcopic results (N = 6) showed the same results between urine and vaginal self-swab samples with regards to HPV DNA positivity.

**Table 3:** HrHPV results and cervical biopsies in women with NILM cytology (n = 33).

CYTOLOGY (n = 33)	Cervical biopsies	HPV DNA results		No. of HPV16/18 positive
		Urine	Vaginal self-swab	
NILM or inflammation	Normal (n = 6)	HPV16 (n = 1) HPV18 (n = 2), HPV18/other (n = 1) Other hrHPV (n = 2)	HPV16 (n = 1) HPV18 (n = 2), HPV18/other (n = 1) Other hrHPV (n = 2)	4
	Chronic cervicitis (n = 16)	Negative (n = 4) Other hrHPV (n = 12)	Negative (n = 2) HPV16 (n = 2) Other hrHPV (n = 12)	0
	CIN1 (n = 11)	HPV16/18 (n = 1) HPV16 (n = 1) HPV18 (n = 1) Other hrHPV (n = 5)* Negative (n = 2)	HPV16/18 (n = 1) HPV16 (n = 1) HPV18 (n = 1) Other hrHPV (n = 6) Negative (n = 2)	3

*Urine not obtained (n = 1).

In this study, only three cases of ASC-US were included. In one woman with both negative urine and vaginal self-swab samples, colposcopic biopsy revealed chronic cervicitis. Meanwhile, in the other two women positive for hr-HPV, colposcopic biopsy revealed CIN3 and CIN1, respectively (Table 4).

**Table 4:** HPV positivity in urine and vaginal samples from women with ASCUS cytology.

Serial No.	Age	CYTOLOGY	Urine	Vaginal self-swab	Colposcopic biopsy
NCC0077	45	ASCUS	negative	negative	Chronic cervicitis
NCC0159	48	ASCUS	HPV18	HPV18	CIN3
NCC0482	30	ASCUS	other	other	CIN1

## Discussion

This pilot study is the first prospective study to use non-invasive, urine and vaginal self-swab sampling for cervical cancer screening at a tertiary hospital in Korea. However, our results could not confirm whether HPV screening based on urine or vaginal swab sampling shows increased sensitivity compared to cytology for detection of CIN2+ or CIN3+ cancer due to the limited sample size: only one participant (1/700) was found to have CIN2+ or CIN3+ disease in this pilot study.

HPV detection rates for the general population of Korea have been found to vary according to age groups, region, socioeconomic status, and methods for detecting HPV DNA, ranging from 10% to 15%^19^. In this study, hrHPV was detected in 6.7% of urine samples and in 9.6% of vaginal self-swab samples. HPV16/18 was detected in 1.5% of urine samples and in 2.0% of vaginal self-swab samples. Other hrHPV was detected in 5.2, and 7.6% of the urine and vaginal self-swab samples, respectively. Although a statistically significant difference in the detection rate of hrHPV was noted between urine and vaginal self-swab samples (p < 0.001), the agreement for HPV 16/18 was relatively high (99.1%, 95%CI 98.1~99.6%), with a kappa of 0.75. Discrepancies arose mainly in the positive samples for hrHPVs other than HPV 16 and 18.

A previous study indicated that the sensitivities for detecting HPV were 96.4% in vaginal self-samples and 83.9% in urine samples relative to cervical samples acquired by a clinician^20^. A recent meta-analysis showed that HPV 16/18 detection in urine has a pooled sensitivity of 73% (56% to 86%) and a specificity of 98% (91% to 100%)^21^. Similar to these studies, for hrHPV detection using Roche_HPV in urine samples we found relative sensitivities of 70.4~79.2% and relative specificities of 93.5~100.0%^18^. Increasing the sensitivity of hrHPV detection in urine samples is needed prior to the use thereof in cervical cancer screening programs^18,22^. One of the reasons for the relatively inferior sensitivity of urine samples in the detection of hrHPV is due to the use of random-void urine samples with no preservatives, instead of an initial stream of urine. Stanczuk *et al*. suggested that the sensitivity of the cobas 4800 HPV test for the detection of CIN2+ could be increased if the cut-off value was increased to 45 PCR cycles^17^. We also showed that RQ-PCR with an adjusted cut-off C_T_ value increased the relative sensitivity of HPV detection in urine samples in our previous study^18^. Thus, if the adjusted cut-off values of RQ-PCR-based tests would be applied for urine samples, detection of HPV DNA using urine samples could be promising for full prospective study for cervical cancer screening.

The reported prevalences of HPV infection in several studies have been found to vary. In several studies, subjects comprised highly-selected, high-risk individuals referred for further evaluation with abnormal cytology in primary cervical screening^23^. Compared to the general population, the prevalence of abnormal cytology (12–83%) or HPV infection (4–91%) in referred populations has been shown to be much higher^24^. Due to overall lower rates of abnormal cytology and lower sexual risk behavior in Korea, a larger sample size than expected may be needed to comprehensively evaluate the effectiveness of self-sampling in detecting CIN2 disease or greater. For a sufficient number of women with CIN2 disease or greater in the ATHENA trial, an estimated 45,000 women were required for analysis: calculations were based on reported rates of ASC-US cytology^25^. The prevalence of ASCUS in the ATHENA study was 4.1%^26^. In this pilot study, however, an unexpectedly low ASCUS rate was discovered, although this finding is similar to rates of abnormal cytological results (0.52%) reported for the national cervical cancer screening program in Korea in 2014^3^. In addition, the number of CIN2 or CIN3 cases included in this pilot study was also unexpectedly low, compared to that in previous studies^27,28^. Thus, a much larger sample size than that in the ATHENA study is needed for definitive study.

In Korea, women aged 30–39 years have had the second-lowest (27.7–44.9%) participation rate in cervical cancer screening, but the highest rate of abnormal results, compared to other age groups, since 2012^4^. This participation rate is much lower than that in Europe^29^ and the US^30^. In addition, cervical cancer incidence rates have been increasing in younger, sexually active groups^4^. For early detection in screening programs, the use of non-invasive self-sampling HPV tests may help with promoting participation among younger women and in preventing cervical cancer in Korea. Indeed. among the participants in the present study, overall satisfaction was 91.4% with the urine sampling and 92.7% with the vaginal self-swab sampling, which was higher than that with clinician-guided cervical swabs (88.1%) (data not shown). Additionally, embarrassment with vaginal self-swabs and with urine sampling was significantly lower than that with clinician-guided Pap smear.

This study had several limitations, including the relatively small sample size. The number of women with ASCUS was very small (n = 3), and only one of the women who underwent colposcopy had a CIN2+ disease. In addition, not all women with NILM underwent colposcopy. Thus, we could not assess the sensitivity of CIN2+ detection by hrHPV DNA testing, although population-based cervical cancer screening samples were collected. In addition, the use of random-void urine samples with no preservatives could compromise the performance of RQ-PCR assays using urine samples.

In summary, Roche_HPV showed relatively good concordance between urine and vaginal self-samples. However, hrHPV DNA detection using urine samples seemed to be inferior to HPV DNA detection using vaginal self-swab samples. A large prospective study should be performed to determine if urine and vaginal self-swab samples may be an effective alternative for HPV detection in women who hesitate to participate in cervical cancer screening programs that use conventional methods.

## Methods

### Participants

To evaluate the performance of Roche_HPV for HPV DNA detection, paired urine and vaginal self-swab samples were collected prospectively from 712 women who visited the National Cancer Center in Korea between July 2016 and November 2016. This study was approved by the Institutional Review Board of the National Cancer Center, Seoul (IRB No. NCC2014-0183 and NCC2015-0066). All women provided informed consent to participate in the study. We confirmed that all experiments were performed in accordance with relevant guidelines and regulations.

### Sample collection, processing, and testing

The research ethics committee of the Institutional Review Board of the National Cancer Center approved the guidelines and regulations for all study methods. The collecting and processing procedure was the same as that described in a previous study^12^. Vaginal swabs were collected via a dry cone-shaped flocked swab (52980 C, FLOQSwabs, Copan Italia, Brescia, Italy) stored in a dry tube.

Study participants also provided urine samples of approximately 30 mL. Before high-risk hrHPV DNA testing using Roche_HPV, each urine sample was washed with Roche Transport Medium. Briefly, 5 mL of each urine sample was mixed with 5 mL of Roche medium and then centrifuged at 3000 rpm for 15 min. After removing the supernatant, the pellet was resuspended in 2 mL of Roche medium and used for HPV testing.

The self-samples were compared to cervical cytology samples, which were collected by a clinician using ThinPrep solution (PreservCyt Solution, Hologic, UK) as per routine protocol in the cancer screening program. Colposcopy was performed and directed biopsies were obtained if clinically indicated. In addition, colposcopy with biopsy was performed for women with hrHPV DNA positive results and, if desired, for those with normal cytological results.

All samples were tested with Roche_HPV based on RQ-PCR as described in a previous study^18,31^ and in accordance with the manufacturer’s instructions. Roche_HPV is a fully automated system for DNA extraction and detection, and provides specific genotype information for HPV types 16 and 18, while concurrently detecting the other 12 hrHPV types in a pooled analysis^12^.

To investigate the participants’ satisfaction with all three sampling methods, we administered a survey questionnaire. Satisfaction and embarrassment were assessed via two respective questions for each sampling method: for example, “Overall, how satisfied were you with the urine sampling?” and “How embarrassed were you with urine sampling?” The items were answered on a 4-point Likert scale, ranging from “very satisfied” to “very unsatisfied.” Overall satisfaction was defined as “Yes” with a Likert score of 1–2 and “No” with a Likert score of 3–4, while embarrassment was defined as “Yes” with a Likert score of 3–4 and “No” with a Likert score of 1–2. In total, 551 participants completed to this questionnaire, and the overall response rate was 78.7%.

### Statistical analyses

Kappa coefficients with 95% confidence intervals (CIs) were calculated to estimate agreement between the results of Roche_HPV tests. McNemar’s test was applied to assess the significance of differences between two correlated proportions. Statistical significance was defined as p < 0.05. All statistical analyses were performed using NCSS software, version 10 (NCSS Statistical Software, Kaysville, UT, USA).
